# Mobile Learning in Medical Education: Quasi-Experimental Realist Evaluation of Usage, Context, and Examination Performance in a Curricular Setting

**DOI:** 10.2196/85892

**Published:** 2026-05-21

**Authors:** Jan Peter Herbert Holzhäuer, Joy Backhaus, Thiên-Tri Lâm, Sarah König

**Affiliations:** 1 Institute of Medical Teaching and Medical Education Research, University Hospital Würzburg, Josef-Schneider-Str. 2/D6, Würzburg, Germany, +49 931 201 55210, +49 931 201 655222; 2 Institute for Hygiene and Microbiology, University of Würzburg, Würzburg, Germany

**Keywords:** medical education, mobile learning, realist evaluation, usage patterns, digital learning tools, technology acceptance, student engagement, educational technology, context factors, equity, artificial intelligence, AI

## Abstract

**Background:**

Mobile learning (mLearning) is widely used in medical education. Previous research has focused on technology acceptance and intervention effectiveness, but rarely on their integration. Using realist evaluation, this study investigated the conditions under which mLearning is adopted and associated with learning-related outcomes in an authentic curricular setting.

**Objective:**

This study aimed to examine how learner context and engagement patterns shape mLearning use and outcomes, while secondarily contextualizing its association with examination performance.

**Methods:**

A quasi-experimental study was conducted among fifth-semester undergraduate medical students at a German medical school across 2 consecutive summer semesters (2023 and 2024). Students were offered a voluntary, app-based mLearning course in microbiology*,* delivered via the eSquirrel platform. The course comprised interactive tasks, incorporating elements of gamification and spaced-repetition features. Data sources included nonreactive in-app usage logs, baseline academic performance data, demographic information, and postsemester questionnaire responses. Usage profiles were derived using cluster analysis. Context-mechanism-outcome patterns were explored by relating app usage status to academic performance and survey responses.

**Results:**

Of the 245 eligible students, 220 (89.8%) participated in the study; 110 (50%) used the app. In 2024, app users (n=64, 58%) initially appeared to outperform nonusers (n=46, 42%) in the oral microbiology examination (mean grade 2.3, SD 1.1 vs 2.8, SD 1.3; *t*_63,0_=1.90; 1-sided *P*=.03). After adjustment, these differences were largely explained by baseline academic performance, with only limited evidence of an independent intervention effect. Cluster analysis of app users identified 3 engagement profiles: continuous low-intensity use (n=60, 54%), increased use before the examination (n=31, 28%), and use at the beginning of the semester (n=19, 17%). Cluster 2 reported the greatest enjoyment, satisfaction, perceived learning gains, and examination performance in microbiology.

**Conclusions:**

Nonreactive in-app usage data provided valuable insights into student engagement. The effectiveness of mLearning was not universal. Examination-oriented use, associated with more strategic and self-regulated study behavior, was linked to more favorable learning outcomes. Future research needs to address equity concerns, as higher-performing students tended to benefit most, as well as explore adaptive, context-sensitive approaches to support diverse learners.

## Introduction

Mobile learning (mLearning) has become a prominent but also debated concept in technology-enhanced education. Definitions of mLearning remain inconsistent and have shifted alongside changing technological and pedagogical paradigms [1-6]. In the early 2000s, mLearning was mostly defined as “learning anytime and anywhere using mobile devices” [7-12]. This device-oriented view initially aimed to distinguish mobile from electronic learning and other stationary formats. With the widespread adoption of smartphones, early visions of an all-in-one mLearning device, always connected, interactive, and multimedia-capable, started to become a reality [13,14]. More recent definitions are increasingly theory-based approaches and adopt learner-centered perspectives. They emphasize context-awareness, personalization, and continuity between physical and digital learning spaces [4,13-24]. In this view, mLearning is no longer merely a technological mode of content delivery, now reflecting the educational dynamics of learning in increasingly mobile, digitally mediated spaces [25-27]. Accordingly, mLearning describes how learners engage with educational content across contexts, locations, and temporal boundaries [20,28-31]. This aligns with contemporary, postpandemic developments in technology-enhanced education research, which increasingly emphasize the social, interactive, and relational dimensions of technology-learner interaction, including artificial intelligence (AI)–supported learning environments [32].

Notably, some scholars argue that mLearning also includes traditional learning activities such as reading a textbook while commuting [28,33-35]. At the same time, rapid technological innovation challenges the generalizability of empirical findings [35]. What was once counted as mLearning, for example receiving an SMS text message from a teacher [14], differs markedly from today’s AI-powered mobile apps, augmented reality tools, or social robots [32]. The contexts in which mLearning has been evaluated are highly diverse [14,15,36]: Existing studies have made valuable contributions by examining a wide range of interventions across educational levels, settings, and technologies. However, the substantial heterogeneity in intervention types, instructional content, outcome measures, and study populations makes it difficult to draw generalizable conclusions. Although the COVID-19 pandemic marked a major surge in mLearning research, it also exposed persistent conceptual and methodological fragmentation in the field, as documented in recent bibliometric syntheses [37]. This fragmentation underscores the need for context-sensitive approaches that move beyond average effects to examine mLearning in authentic curricular settings.

Although much of the literature is optimistic [38-43], mLearning has not delivered the anticipated educational transformation [44]. Accordingly, more context-sensitive research is needed to understand how and under what conditions mLearning can be effectively integrated into higher education [45]. This challenge is particularly pressing in undergraduate medical education [46], in which curricula are shifting toward a hybrid and individualized format and in a broader move from knowledge transmission to competency-based learning [47,48]. As students are expected to develop professional competencies rather than merely acquire facts, a didactic gap emerges: foundational theoretical knowledge must be acquired through self-directed learning in informal contexts. mLearning may help bridge this gap by supporting learners’ self-directed and autonomous learning, and by linking theoretical knowledge with the development of applied competencies [49-51]. By providing content access “anytime and anywhere,” it can generate informal learning episodes that complement clinical training and enhance readiness for professional practice. Consistently, informal learning spaces are gaining importance [52]. Accordingly, mLearning can contribute to more integrated, context-sensitive learning trajectories in medical education [53].

Within mLearning research, 2 major lines of inquiry have received particular attention [54]: (1) studies examining the overall effectiveness of mLearning interventions and (2) studies investigating psychological and contextual factors influencing learners’ uptake. However, these perspectives have rarely been integrated in authentic curricular settings. As a result, there is limited understanding of how learners engage with mobile tools, how contextual factors shape this engagement, and how engagement relates to academic outcomes. To address this gap, we applied the realist evaluation (RE) framework [55] to examine the complex interplay. Using the context-mechanism-outcome (CMO) model [56,57], we conceptualize mLearning as a contingent process in which learner-specific contexts (C) activate engagement mechanisms (M) that, in turn, have measurable outcomes (O).

Driven by an initial program theory, we assumed that heterogeneous curricular contexts shape how learners engage with the intervention. We hypothesized that structured mLearning resources activate different motivational and self-regulatory processes depending on prior academic performance and learner characteristics.

Accordingly, this study pursued 2 main objectives with a predominantly exploratory orientation: (1) to examine whether voluntary participation in mLearning was associated with examination performance within a routine medical curriculum and (2) to explore how learner contexts influenced engagement patterns by deriving usage profiles from in-app engagement data and relating them to contextual variables and academic outcomes.

## Methods

### Evaluation Setting and Methodical Approach

An RE design [53] was used to identify CMO patterns under routine curricular settings. Accordingly, no randomization or blinding was applied. The evaluation was conducted at a German medical school (6-year curriculum). Within the faculty-wide digitalization initiative WueMedica, a general mLearning module was developed and implemented across multiple disciplines. In this study, we focused on its use in the fifth-semester microbiology course, comprising bacteriology, parasitology, mycology, and virology. The subject-specific component of the module was delivered via the eSquirrel app (eSquirrel GmbH). In addition to a voluntary lecture series and mandatory weekly laboratory sessions, mLearning was offered as a supplementary learning resource. Teaching staff delivered the standard curriculum independently, and no direct contact occurred between the study team and students.

### Intervention

The intervention consisted of a voluntary app-based course designed to support examination preparation alongside the curriculum. Content was developed by trained student assistants in collaboration with faculty experts and was based on official course materials, the Nationaler Kompetenzbasierter Lernzielkatalog Medizin (the German national competency-based learning objectives catalog) [58], and standard textbooks. The app-based course was organized into chapters with interactive tasks (such as multiple-choice, matching, and fill-in-the-blank formats) and supplemented by images and links to relevant textbook chapters. Gamification features such as point scoring, a leaderboard, push notifications, and an adaptive spaced-repetition algorithm were included. The course comprised 88 chapters in 2023 and was expanded to 138 in 2024.

### Ethical Considerations and Data Protection

The study was approved by the local independent review board (ref 20230217 01 and ref 202580-ka) and conducted in accordance with the European Union General Data Protection Regulation. All study data were stored on secure institutional servers of the University Hospital. The app complied with General Data Protection Regulation requirements and supported cross-platform and offline use.

Students were informed about the study during an in-person session at the beginning of the semester and provided written informed consent. Consent included permission to link the pseudonymized student identifiers with examination results and study data temporarily. Access to the mLearning app was provided via the Moodle-based institutional learning management system (Moodle Pty Ltd, West Perth, Australia) and required an official university email address. Student IDs were used solely for data linkage purposes and removed from the merged dataset prior to analysis. Data from students who used the app but did not consent to participation were excluded. It was determined in 2023 that multiple registrations were technically possible, but these were effectively prevented in 2024 through email verification and ID matching. Participation was voluntary and had no impact on students’ courses, assessments, or academic progression.

### Study Design and Data Sources

The evaluation spanned 2 consecutive summer semesters (2023 and 2024). Eligible participants were students enrolled in the microbiology course and taking the oral examination at the end of the course for the first time. Repeat examinees or students without formal registration were excluded.

Data were collected from four sources:

1. App usage data (nonreactive) as in-app tasks within chapters representing activity (chapters completed), task success (points earned), and learning efficiency (chapters per unit time).
2. An online questionnaire administered at the end of the semester via EvaSys (evasys GmbH, Lüneburg, Germany) assessing demographics (age and gender), app usage, and learning-related characteristics; 19 items adapted from past studies (the unified theory of acceptance and use of technology [59] and the Revised Two-Factor Study Process Questionnaire [60], as well as studies on technology-enabled formative assessment in medical education [61] and the use of unsupervised online quizzes in medical physiology [62]); and open-ended questions on user satisfaction. The questionnaire was originally developed in German and then translated into English.
3. End-of-course oral microbiology examination grades in 2024, obtained at the end of the fifth semester; grades ranged from 1 (very good) to 5 (failed).
4. Percentage scores from the written part of the German first national licensing examination (Staatsexamen M1) after the fourth semester, served as an indicator of prior academic performance.

In 2023, 117 students qualified for participation. On the basis of an expected 80% (94/117) participation rate, the study was powered to detect a moderate effect size (Cohen *d*=0.58) at α=.05 with 80% power.

### Data Analysis

Standardized usage and demographic variables (z-scores, calculated as z=(X–M)/SD, X as individual values, means, and SDs) were entered into a 2-step cluster analysis (log-likelihood distance and the Bayesian information criterion) to identify user profiles. Students were categorized according to app usage status (nonusers vs app users). App users were further grouped into usage clusters based on engagement patterns. All groups were compared with respect to survey responses and performance outcomes.

Survey data were analyzed using principal component analysis with pairwise deletion for missing values. Components with eigenvalues >1 were extracted and subjected to oblique (Oblimin) rotation. Reliability was assessed with Cronbach α, and component scores were linearly rescaled to a 1-to-5 scale to ease interpretation. Variance contributions of the final scales were approximated using regression-based scores from the unrotated solution.

For the 2024 data, an analysis of covariance was conducted to test for any potential intervention effect. The written M1 examination score as a percentage was used as the academic baseline, and the analysis examined whether oral microbiology examination results deviated significantly from this baseline. Data from 2023 were not included due to methodological limitations. These included incomplete coverage of exam-relevant chapters in the app and the inability to identify app users clearly.

All analyses were conducted using SPSS Statistics (version 29.0.0.0; IBM Corp) at a 95% significance level, applying Welch *t* tests (2-tailed) for unequal variances [63,64] and standard *t* tests when variances were equal, with 2-sided *P* values reported unless otherwise specified.

Reporting followed the CONSORT-EHEALTH (Consolidated Standards of Reporting Trials of Electronic and Mobile Health Applications and Online Telehealth V.1.6.1; Checklist 1) checklist for mobile health interventions [65] and the RAMESES-II (Realist and Meta-narrative Evidence Syntheses: Evolving Standards-2; Checklist 2) standards for REs [66].

## Results

### Participant Flow and Sample Characteristics

**Figure 1.**
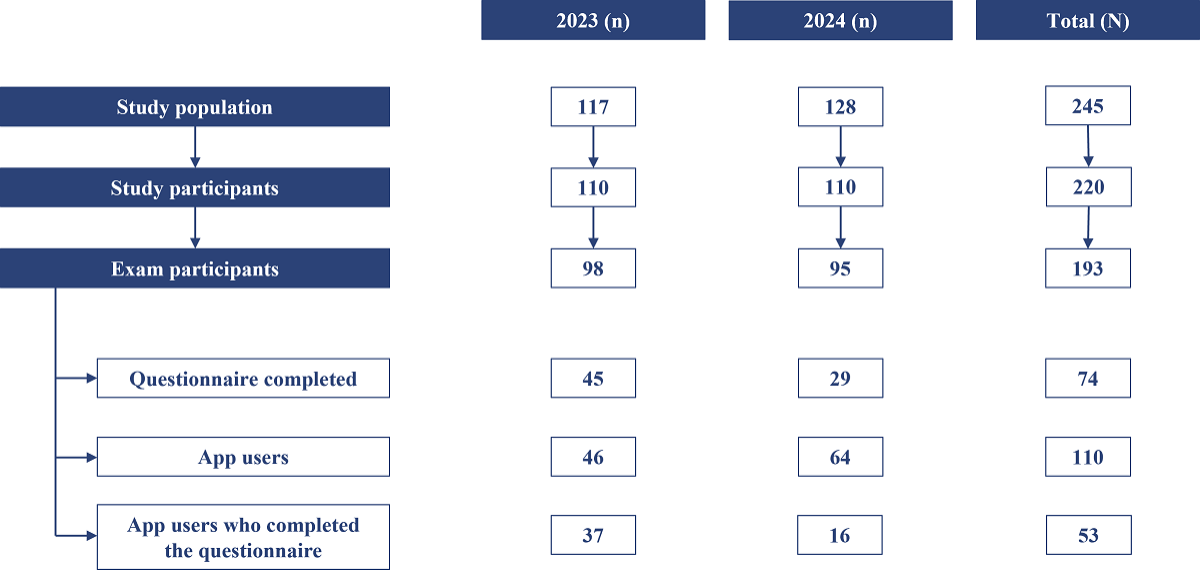
Flowchart of participant recruitment and data availability.

There were 245 eligible students, of whom 220 (89.8%) participated. Participant recruitment and data availability are summarized in Figure 1. Participants were categorized according to user status, with 110 app users and 110 nonusers. The survey produced 74 valid responses out of 220 participants, corresponding to an overall response rate of 33.6%. In total, 53 participants (24.1%) both used the app and completed the survey. Demographic characteristics were representative of the student population (study participants: 70% female; mean age 23.1 years, SD 3.1 years and overall study population: 69% female; mean age 23.9 years, SD 3.3 years). No significant differences were observed between semesters. Baseline characteristics of participants are provided in Multimedia Appendix 1.

### App Usage Clusters

**Figure 2.**
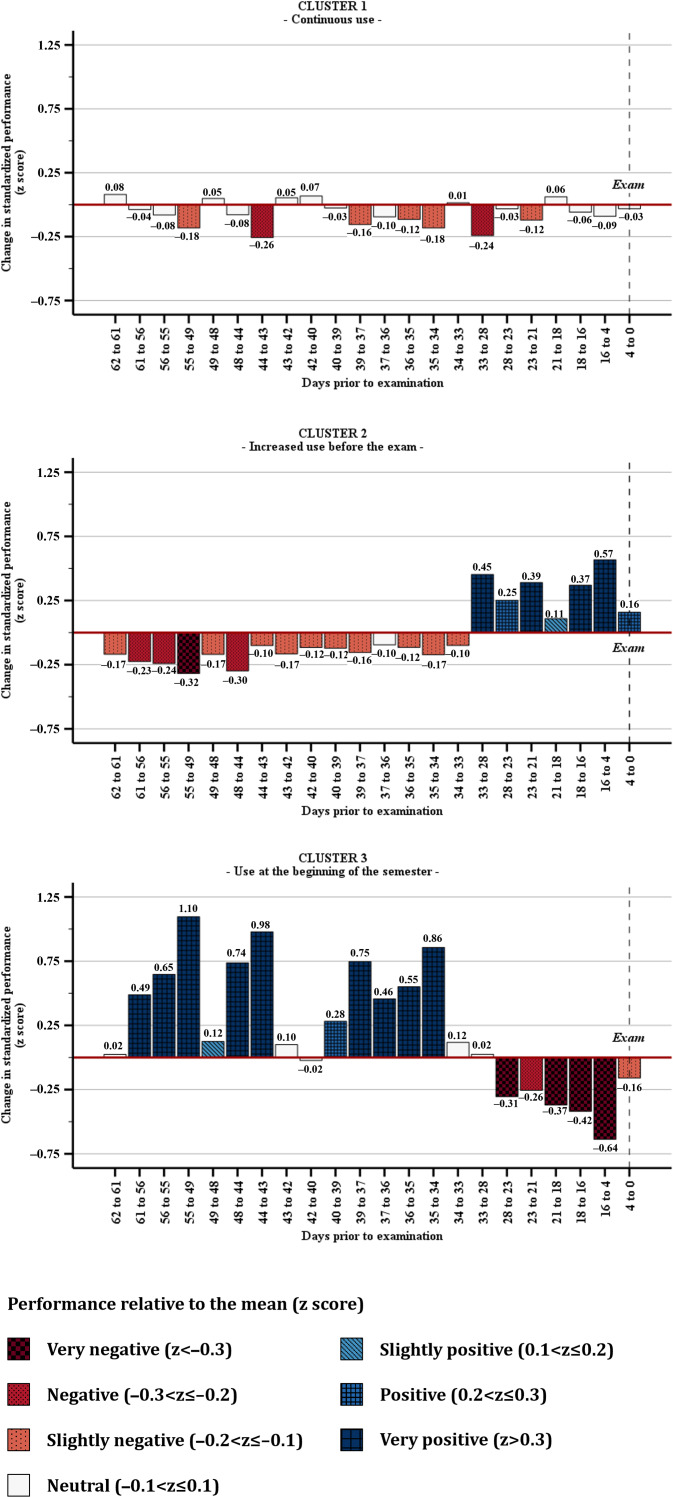
Longitudinal z-standardized engagement trajectories (completed in-app tasks within chapters) across app-user clusters throughout the semester.

Cluster analysis (average silhouette coefficient=0.40) of the 110 app users identified 3 usage clusters that differed primarily in the timing and intensity of engagement throughout the semester. Figure 2 illustrates the corresponding longitudinal z-standardized engagement trajectories, with user performance defined as successfully completed in-app tasks within chapters.

Cluster analysis revealed three distinct usage patterns:

- Cluster 1 (“continuous low-intensity use,” n=60) exhibited regular activity across the semester (active on 66.6% of days) but low content completion (26.5% of in-app tasks in chapters), with engagement largely confined to rare content repetitions (78.4% of app chapters repeated only once) and the lowest task success rate (11.6%).
- Cluster 2 (“increased use before the examination,” n=31) combined a lower proportion of active days (48.1% of days) with high content completion (80.9%), a more balanced distribution across content repetitions, and moderate task success (45.4%), with engagement intensifying markedly shortly before the examination.
- Cluster 3 (“use at the beginning of the semester,” n=19) demonstrated the highest overall engagement (active on 87.2% of days), substantial content completion (67.3%), and the highest content repetition intensity (35.6% of app chapters repeated 4 times), but engagement peaked early and declined approximately 4 weeks before the examination period.

No significant differences in age or gender were observed across clusters.

The bar charts illustrate changes in standardized performance (*z* scores) over the course of the semester. Positive values indicate above-average gains relative to all users; negative values indicate below-average gains. For example, between days 39 and 37 before the examination, cluster 3 users achieved gains around 0.75 SD above the mean.

### Oral Examination Performance According to App Usage Status

For the 2024 cohort, end-of-course oral examination results were available for 95 participants; 15 students (13.6%) were excused for medical reasons. Grades ranged from 1 (excellent) to 5 (failed; Table 1). App users (n=64) achieved better examination results than nonusers (n=46; mean grade 2.3, SD 1.1 vs 2.8, SD 1.3; 1-sided Welch *t*_63,0_=1.90; *P*=.03). Among app users, cluster 2 (n=20) presented the best performance (mean 2.1, SD 0.9; *P*=.008 vs nonusers), whereas cluster 1 (n=34; mean 2.4, SD 1.1; *P*=.09) and cluster 3 (n=10; mean 2.5, SD 1.3; *P*=.26) did not differ significantly from nonusers. A higher proportion of nonusers than users were excused from the examination (24% vs 6%). No statistically significant differences were observed between the 3 app-user clusters.

**Table 1.** Oral examination grades in microbiology stratified according to app usage status and usage clusters (N=110).

	Total	Nonusers (n=46)	App users			
			Total (n=64)	Cluster 1 (n=34)	Cluster 2 (n=20)	Cluster 3 (n=10)
Results, mean (SD)	2.5 (1.2)	2.8 (1.3)	2.3 (1.1)	2.4 (1.1)	2.1 (0.9)	2.5 (1.3)
Grade 1, n (%)	19 (17)	5 (11)	14 (22)	7 (21)	5 (25)	2 (20)
Grade 2, n (%)	36 (33)	12 (26)	24 (38)	12 (35)	8 (40)	4 (40)
Grade 3, n (%)	21 (19)	7 (15)	14 (22)	8 (24)	4 (20)	2 (20)
Grade 4, n (%)	12 (11)	7 (15)	5 (8)	3 (9)	1 (5)	1 (10)
Failed, n (%)	7 (6)	4 (9)	3 (5)	2 (6)	N/A^a^	1 (10)
Excused, n (%)	15 (14)	11 (24)	4 (6)	2 (6)	2 (10)	N/A

a N/A: not applicable.

### Questionnaire Results: Psychometric Properties and Scale Structure

Sampling adequacy was acceptable (Kaiser-Meyer-Olkin test=0.750; Bartlett test *P*<.001). Principal component analysis initially identified 6 scales explaining 74.3% of the variance. Scales 3 to 5 were excluded due to low internal consistency (Cronbach *α*=0.46-0.09) and content overlap. Scale 1 was divided into *learning outcome* and *user experience* in line with the theoretical framework [67,68], with 2 items reassigned to enhance conceptual fit. Scale 2 was retained without modification. Three final scales were identified: *learning outcome* (perceived gains in learning efficiency, comprehension, and long-term retention), *user experience* (usability, enjoyment, motivational impact, feedback on progress, curricular relevance, and willingness to recommend), and *study motivation* (general motivation, avoidance of superficial learning, openness to engaging topics, and preference for self-testing) with item loadings depicted in Table 2. Together, the 3 scales required no further rotation and explained 50.2% of the total variance (Table 3).

**Table 2.** Principal component analysis of questionnaire data: item loadings and internal consistency.

Item	Item loading		
	Learning outcome	User experience	Study motivation
4.1: mLearning with WueMedica enhances my learning outcomes.	0.92	—^a^	—
4.2: mLearning with WueMedica makes my studying more effective.	0.92	—	—
4.3: mLearning with WueMedica improves my understanding of the learning topics.	0.80	—	—
4.4: mLearning with WueMedica helps me retain what I have learned for a longer period.	0.59	—	—
5.1: mLearning with WueMedica is intuitive.	—	0.57	—
5.2: mLearning with WueMedica excites me.	—	0.87	—
5.3: mLearning with WueMedica motivates me to study more than I did before.	—	0.60	—
5.4: mLearning with WueMedica is enjoyable.	—	0.70	—
5.5: mLearning with WueMedica provides me with feedback on my learning progress.	—	0.49	—
5.6: mLearning with WueMedica helps me identify my strengths and weaknesses in learning.	—	0.46	—
5.7: mLearning with WueMedica adds value to the curricular teaching.	—	0.82	—
6.1^b^: Studying sometimes gives me a sense of deep satisfaction.	—	—	–0.27
6.2: My goal is to pass the course with as little effort as possible.	—	—	0.53
6.3: I only study the topics that are relevant for the exam.	—	—	0.73
6.4: Any topic can become interesting to me once I am engaged with it.	—	—	–0.44
6.5: I learn some things by repeating them until I have memorized them, even if I do not understand them.	—	—	0.68
6.6: I believe that I can pass most exams by memorizing the key points rather than understanding them.	—	—	0.68
6.7: I like to test myself on the key topics until I fully understand them.	—	—	–0.65
6.8: I will recommend mLearning with WueMedica to others.	—	0.90	—

a Loadings below the reporting threshold.

**Table 3.** Cronbach α and explained variance of the principal component analysis–derived scales.

	Learning outcome	User experience	Study motivation
Cronbach α	0.90	0.82	0.67
Explained variance (%)	22.8	13.4	14.0

Detailed results according to usage status and usage clusters are reported in Multimedia Appendix 2.

### Questionnaire Results: Scale Scores According to App Usage Status

We compared app users and nonusers at both the scale and item level. We observed no statistically significant differences at the scale level. At the item level, however, app users consistently rated more favorably, most notably with regard to enjoyment.

Cluster 1 was not found to have any advantage over nonusers on any scale. At the item level, enjoyment was the only aspect rated higher than by nonusers.

Cluster 2 exhibited the most favorable self-reported learning experience. Compared with nonusers, this cluster scored significantly higher on *learning outcome* and *user experience* scales. Furthermore, *learning outcome* scores were also higher than those of cluster 1. No differences were observed for *study motivation*. At the item level, cluster 2 exhibited consistently higher ratings across multiple items related to perceived learning gains, enjoyment, feedback on learning progress, satisfaction with studying, and willingness to recommend the app.

Cluster 3 did not differ from nonusers or other clusters for outcomes at the scale level. At the item level, enjoyment was again rated more favorably than by nonusers. Descriptive questionnaire results are provided in Multimedia Appendix 2.

### Open-Ended Responses

Open-ended responses are provided as tabulated summaries in Multimedia Appendix 3. Among nonusers and cluster 1, the most frequently reported themes were time constraints and a perceived misalignment between the app content, predominantly consisting of selected-response formats, and the oral examination format. Participants from clusters 2 and 3 more frequently highlighted motivational aspects and emphasized the app’s contribution to learning the subject of microbiology. In these clusters, positive experiences related to enjoyment, perceived progress, and engagement were more prominent. For example, there were frequent references to gamification features such as affirmation through comparative rankings.

### Baseline-Controlled Examination Performance According to App Usage Status

Analyses focused on the summer semester of 2024, including 35 nonusers and 60 app users distributed across 3 usage clusters. The written M1 examination percentage score served as the primary standardized measure of prior academic ability (Table 4).

**Table 4.** Examination performance by app usage status and usage clusters^a^ (N=210^b^).

	Students, n	Microbiology examination oral grade, mean (SD)	National licensing examination written score (%), mean (SD)
Eligible population	210	2.5 (1.2)	74.0 (8.5)
Total participants	189	2.5 (1.2)	73.9 (8.5)
Summer 2023	94	2.5 (1.2)	75.6 (8.8)
Summer 2024	95	2.5 (1.2)	72.2 (7.8)
Nonusers	35	2.8 (1.3)	70.2 (8.1)
Users	60	2.3 (1.1)	73.4 (7.5)
Cluster 1	32	2.4 (1.1)	72.3 (6.8)
Cluster 2	18	2.1 (0.9)	73.8 (8.2)
Cluster 3	10	2.5 (1.3)	76.6 (8.1)

a Grades for both microbiology and national licensing examination (M1) range from 1 (excellent) to 5 (failed). The written M1 examination results are reported as percentage scores, calculated as the proportion of points achieved relative to the maximum possible score.

b Data are presented for the 210 participants with available M1 scores; the total study sample comprised 220 individuals.

At this baseline, users had slightly higher written M1 examination scores than nonusers (73.4% vs 70.2%; *P*=.04). Comparing usage clusters, only cluster 3 scored significantly higher than nonusers (76.6% vs 70.2%; 1-sided *P*=.02), while cluster 1 scores did not differ significantly from those of nonusers (72.3% vs 70.2%; 1-sided *P*=.12). Cluster 2 exhibited a nonsignificant trend toward higher performance (73.8% vs 70.2%; 1-sided *P*=.06).

Within-group analyses of oral microbiology grades relative to the written M1 baseline revealed significant improvement for nonusers (*P*=.04), cluster 1 (*P*=.001), and cluster 2 (*P*=.01). In contrast, cluster 3, despite the highest written M1 examination score (76.6%), did not exhibit any significant improvement (*P*=.34). Among users, cluster 3 had the lowest oral microbiology grade (mean 2.5), whereas nonusers scored slightly lower (mean 2.8). A significant interaction effect between cluster 2 and nonusers was observed for oral microbiology grades (1-sided *P*=.04).

Written M1 examination scores predicted oral microbiology grades significantly (*P*=.006; η_p_²=0.08). After analysis of covariance adjustment, the group×examination interaction for users versus nonusers remained statistically significant (*P*=.03; η_p_²=0.05), whereas cluster interaction effects were not significant (*P*=.23; η_p_²=.05). Effect sizes were small.

## Discussion

### Integration of Findings Within Existing Literature

Established models of mLearning emphasize that learning outcomes emerge from the interaction between learning context, technological affordances, and individual learner characteristics, rather than from the technology alone [17,19,20]. However, evidence derived from authentic curricular settings remains limited. The primary aim of this study was not to demonstrate the effectiveness of a specific mLearning intervention. Instead, we examined how learner characteristics and contextual conditions shape engagement and learning outcomes when mLearning is offered as a voluntary supplement within a routine medical curriculum [16,17]. By integrating effectiveness-related outcomes with technology acceptance perspectives within a RE framework, we moved beyond average effects. This enabled us to identify CMO configurations that explain heterogeneity in engagement patterns and learning outcomes (Multimedia Appendix 4) [55].

A central finding of the study was that prior academic performance remained the strongest predictor of examination scores, while the independent effect of app use was small after statistical adjustment. Rather than constituting a limitation, this finding represents a key empirical result. Students with higher baseline academic performance were more likely to engage with the app, reported more favorable learning experiences, and achieved better outcomes. This pattern is consistent with previous research illustrating that academically stronger students tend to display greater self-regulatory capacity, stronger goal orientation, and a higher propensity to engage with optional learning resources [45,69-71]. Accordingly, we found no evidence that the mLearning intervention compensates for lower baseline academic performance. This raises important equity concerns when mLearning is offered as a voluntary supplement, as it may preferentially benefit academically stronger students and reinforce existing differences [72]. This observation aligns with broader discussions on digital inequities in health professions education, in which students with weaker baseline skills or fewer resources are at risk of being left behind [73-75].

Self-selection provides valuable insight in this setting and should not be regarded solely as a source of bias. However, it limits causal inference regarding any independent intervention effect. At the same time, it directly addresses the core question of this study: Who engages with mLearning under routine curricular settings? It also helps explain why mLearning tools may fail to benefit those learners who arguably need them most. This gap is reinforced by the fact that most existing studies report average effects without stratifying outcomes according to baseline performance, leaving equity-related questions largely unaddressed [42,76].

The cluster analysis further illustrated substantial heterogeneity in engagement trajectories, reinforcing evidence that mLearning use is highly individualized [77]. Neither sporadic and low-intensity use nor early but temporary engagement was associated with favorable outcomes. In contrast, students who integrated the app strategically into existing study routines and used it in an examination-oriented manner exhibited the most favorable profiles. These findings indicate that mLearning does not compensate for absent or inconsistent learning strategies, but rather amplifies existing ones [78]. Instructional features such as spaced-repetition, feedback, or gamification should not be interpreted as mechanisms in themselves, but as affordances that may activate motivational, metacognitive, and self-regulatory processes in specific learner groups [69,77]. Consistent with the RE perspective, mechanisms emerge from the interaction between learner dispositions and contextual conditions, which may render identical features effective for some learners while remaining largely ineffective for others [55,56,60].

In contrast to much of the prior technology acceptance research [59,79-81], which has predominantly emphasized perceived usefulness as the key driver of adoption, our findings indicate that enjoyment emerged as a more robust correlate of sustained engagement and positive learning experiences [76,80,82,83]. In addition, intensive and strategic users were consistently more satisfied with their learning experience. This suggests that affective components play a central role in learners’ intention to engage with mLearning tools, particularly when use is voluntary. In this context, the prominent role of enjoyment highlights the importance of addressing affective and cognitive dimensions of learning [77,80,84].

### Theoretical and Practical Implications

Our findings indicate that mLearning effectiveness is context-dependent rather than technology-driven. Favorable outcomes were observed linked to strategic integration into existing study routines. Accordingly, commonly used instructional elements such as spaced repetition [85-87], gamified feedback [88-90], just-in-time learning [91,92], and microlearning [93-95] should be understood as supportive conditions rather than universally effective components. For curriculum designers, mLearning needs to be embedded within structured learning pathways. Evidence suggests that didactic scaffolding (clear pedagogy, feedback, and structured learning activities) [96] and systemic monitoring of engagement patterns [97] can substantially strengthen mLearning. In addition, learning analytics or AI-supported personalization may help address learner heterogeneity by enabling more adaptive and context-sensitive learning pathways [98-101].

To further operationalize these principles, implementation strategies should target both initial uptake and sustained engagement. First, low-threshold access and structured onboarding processes (eg, seamless integration into learning management systems, single-sign-on, and visible entry points such as QR codes) are essential to reduce barriers to participation. Second, curricular alignment and active implementation strategies**,** including faculty endorsement and in-app prompts such as push notifications, can reinforce learning routines and support sustained, self-regulated learning engagement [39,102]. Third, interventions should explicitly address both perceived usefulness and affective engagement, as both are critical determinants of adoption and continued use in voluntary learning contexts [103]. Fourth, content should follow microlearning principles, using small, manageable units; intuitive navigation; and adaptive difficulty to support learners with different prior knowledge and promote equitable participation [92,95]. Finally, gamification elements (eg, leaderboards, badges, and progress indicators) can support engagement by making learning progress visible, as implemented in the app used in this study.

### Limitations

The quasi-experimental design and voluntary app use limit causal inference, as observed differences in academic outcomes cannot be attributed to the intervention alone. In addition, the absence of randomization and the reliance on observational data restrict conclusions regarding independent intervention effects. Self-selection by higher-performing students precludes attribution of observed differences in academic outcomes to the intervention alone. Within an explanatory framework, however, this self-selection reflects authentic adoption behavior and therefore constitutes a meaningful finding rather than a methodological flaw.

Further limitations include potential expectancy effects, the timing of the postexamination survey, unmeasured learner characteristics (eg, self-regulated learning strategies or time management skills), and limited statistical power in subgroup analyses. Exactly half of the 220 participants used the app, but only 33.6% (n=37) completed the questionnaire. This potential nonresponse bias limits the generalizability of conclusions linking self-reported evaluations to actual usage behavior. The exclusive reliance on quantitative data further constrained interpretive depth and limited insight into learners’ subjective experiences and decision-making processes.

### Future Directions

Future research should integrate summative academic outcomes with longitudinal analyses of engagement behavior to examine how learner characteristics and curricular embedding jointly shape mLearning use and effectiveness.

Prior academic performance proved to be the strongest predictor of engagement in our study. However, other potentially relevant factors were not assessed, such as digital literacy, personality traits, or interest in the subject, which may partly explain cluster formation and differential usage patterns [104-107]. These unmeasured characteristics may influence both students’ interaction with mLearning interventions and the resulting learning outcomes, thereby contributing to the heterogeneous engagement patterns observed. Consequently, future research should systematically investigate these variables, ideally using mixed methods designs that combine quantitative usage tracking with qualitative insights into learners’ intentions and experiences.

Learning analytics and AI-enabled personalization can help address differences between learners. One example of this is providing feedback on engagement and learning behavior to support more individualized learning [108,109] However, their potential to support learners with lower baseline academic performance requires careful empirical evaluation.

Overall, this study highlights the importance of examining mLearning as a situated educational practice, emphasizing the need for designs and evaluations that reflect the complexity of students’ attitudes toward and actual use of mLearning within their learning contexts.

## Supplementary material

10.2196/85892 Multimedia Appendix 1 Baseline characteristics of participants according to semester, including demographics, app usage, questionnaire response, and examination participation (N=220).

10.2196/85892 Multimedia Appendix 2 Questionnaire results: factor scores and item-level ratings according to usage status and usage clusters (N=74).

10.2196/85892 Multimedia Appendix 3 Open-ended responses.

10.2196/85892 Multimedia Appendix 4 Context-mechanism-outcome configurations.

10.2196/85892 Checklist 1 CONSORT-EHEALTH checklist.

10.2196/85892 Checklist 2 RAMESES-II checklist.

## Abbreviations

AI: artificial intelligence
CMO: context-mechanism-outcome
mLearning: mobile learning
RE: realist evaluation
