# Supplementary material for: Mobile Learning in Medical Education: Quasi-Experimental Realist Evaluation of Usage, Context, and Examination Performance in a Curricular Setting
Source: JMIR Med Educ. 2026 May 21;12:e85892. doi: 10.2196/85892 (PMC13193576; doi:10.2196/85892)
Supplement: Multimedia Appendix 1 [file mededu-v12-e85892-s001.docx]

**Appendix 2.** Baseline characteristics of participants according to semester, including demographics, app usage, questionnaire response, and examination participation (N=220)^a^.

| **Category** |  | **Semester** | | ***P* value** |
| --- | --- | --- | --- | --- |
|  |  | **2023** | **2024** |  |
|  |  |  |  |  |
| **Participants** | n (%) | 110 (50) | 110 (50) |  |
|  | Gender, female, n (%) | 80 (72.7) | 74 (67.3) | .38 |
|  | Age, mean (SD), y | 23.0 (3.0) | 23.2 (3.2) | .70 |
| App Usage | n (%) | 46 (41.8) | 64 (58.2) |  |
|  | Gender, female, n (%) | 31 (67.4) | 47 (7.51) | .50 |
|  | Age, mean (SD), y | 23.5 (3.3) | 23.1 (3.3) | .52 |
| Questionnaire | n (%) | 49 (66.2) | 25 (33.8) |  |
|  | Gender, female, n (%) | 36 (73.5) | 16 (64) | .42 |
|  | Age, mean (SD), y | 23.0 (3.1) | 22.7 (2.3) | .60 |
| Examination | n (%) | 98 (50.8) | 95 (49.2) |  |
|  | Gender, female, n (%) | 72 (73.5) | 61 (64.2) | .17 |
|  | Age, mean (SD), y | 22.9 (3.0) | 23.3 (3.3) | .42 |

^a^Participants of both intervention semesters 2023 and 2024.
