# Supplementary material for: Mobile Learning in Medical Education: Quasi-Experimental Realist Evaluation of Usage, Context, and Examination Performance in a Curricular Setting
Source: JMIR Med Educ. 2026 May 21;12:e85892. doi: 10.2196/85892 (PMC13193576; doi:10.2196/85892)
Supplement: Multimedia Appendix 2 [file mededu-v12-e85892-s002.docx]

**Appendix 3.** Questionnaire results: factor scores and item-level ratings according to usage status and usage clusters (N=74^a^).

| **Item** |  | **Total** | **Non-Users** | **App Users** | | | |
| --- | --- | --- | --- | --- | --- | --- | --- |
|  |  |  |  | **Total** | **C1** | **C2** | **C3** |
|  |  |  |  |  |  |  |  |
| **LO** | n | 68 | 15 | 53 | 23 | 17 | 13 |
|  | M(SD) | 2.9 (1.1) | 2.9 (1.5) | 2.9 (1.1) | 3.1 (1.0) | 2.8 (1.3) | 2.7 (0.9) |
|  | *P^b^* |  | .16 | | .048 | | |
| **UE** | n | 70 | 16 | 54 | 24 | 17 | 13 |
|  | M(SD) | 2.5 (0.9) | 2.5 (1.1) | 2.5 (0.9) | 2.7 (1.0) | 2.4 (0.9) | 2.3 (0.7) |
|  | *P* |  | .054 | | .26 | | |
| **SM** | n | 73 | 19 | 54 | 24 | 17 | 13 |
|  | M(SD) | 3.0 (0.9) | 3.0 (0.7) | 3.0 (0.9) | 3.0 (0.9) | 3.1 (1.1) | 2.9 (1.0) |
|  | *P* |  | .47 | | .92 | | |
|  |  |  |  |  |  |  |  |
| **4.1** | n | 68 | 15 | 53 | 23 | 17 | 13 |
|  | M(SD) | 3.7 (1.1) | 3.1 (1.5) | 3.8 (0.9) | 3.5 (0.9) | 4.3 (0.8) | 3.7 (0.8) |
|  | *P* |  | .062 | | .011 | | |
| **4.2** | n | 68 | 15 | 53 | 23 | 17 | 13 |
|  | M(SD) | 3.5 (1.2) | 3.2 (1.5) | 3.6 (1.0) | 3.4 (1.1) | 3.9 (1.0) | 3.4 (1.0) |
|  | *P* |  | .21 | | .16 | | |
| **4.3** | n | 68 | 15 | 53 | 23 | 17 | 13 |
|  | M(SD) | 3.4 (1.2) | 3.3 (1.6) | 3.5 (1.1) | 3.1 (1.1) | 3.7 (1.1) | 3.8 (0.8) |
|  | *P* |  | .32 | | .071 | | |
| **4.4** | n | 66 | 14 | 52 | 22 | 17 | 13 |
|  | M(SD) | 3.6 (1.2) | 3.3 (1.4) | 3.7 (1.1) | 3.6 (1.1) | 4.1 (1.0) | 3.5 (1.1) |
|  | *P* |  | .14 | | .31 | | |
|  |  |  |  |  |  |  |  |
| **5.1** | n | 67 | 13 | 54 | 24 | 17 | 13 |
|  | M(SD) | 4.1 (0.9) | 4.2 (0.8) | 4.1 (0.9) | 4.0 (0.9) | 4.1 (0.7) | 4.1 (1.1) |
|  | *P* |  | .38 | | .97 | | |
| **5.2** | n | 68 | 14 | 54 | 24 | 17 | 13 |
|  | M(SD) | 3.6 (1.1) | 3.3 (1.3) | 3.7 (1.0) | 3.4 (1.1) | 3.9 (1.0) | 3.9 (0.8) |
|  | *P* |  | .17 | | .28 | | |
| **5.3** | n | 69 | 15 | 54 | 24 | 17 | 13 |
|  | M(SD) | 3.3 (1.2) | 2.9 (1.3) | 3.4 (1.2) | 3.1 (1.1) | 3.6 (1.3) | 3.5 (1.1) |
|  | *P* |  | .13 | | .42 | | |
| **5.4** | n | 67 | 14 | 53 | 23 | 17 | 13 |
|  | M(SD) | 3.8 (1.1) | 3.0 (1.2) | 4.0 (0.9) | 3.9 (1.1) | 4.2 (0.7) | 3.9 (0.6) |
|  | *P* |  | .007 | | .56 | | |
| **5.5** | n | 67 | 14 | 53 | 23 | 17 | 13 |
|  | M(SD) | 3.8 (1.1) | 4.0 (1.0) | 3.7 (1.1) | 3.8 (0.9) | 3.7 (1.3) | 2.53.5 (1.1) |
|  | *P* |  | .15 | | .62 | | |
| **5.6** | n | 65 | 12 | 53 | 23 | 17 | 13 |
|  | M(SD) | 3.5 (1.1) | 4.0 (1.0) | 3.3 (1.0) | 3.4 (0.9) | 3.3 (1.2) | 3.3 (1.2) |
|  | *P* |  | .024 | | .95 | | |
| **5.7** | n | 67 | 14 | 53 | 23 | 17 | 13 |
|  | M(SD) | 4.0 (1.0) | 3.6 (1.4) | 4.1 (0.9) | 3.9 (1.0) | 4.3 (0.9) | 4.0 (0.7) |
|  | *P* |  | .12 | | .38 | | |
|  |  |  |  |  |  |  |  |
| **6.1** | n | 73 | 19 | 54 | 24 | 17 | 13 |
|  | M(SD) | 3.5 (1.1) | 3.2 (0.8) | 3.7 (1.2) | 3.6 (1.2) | 3.8 (1.0) | 3.5 (1.3) |
|  | *P* |  | .027 | | .70 | | |
| **6.2** | n | 72 | 19 | 53 | 23 | 17 | 13 |
|  | M(SD) | 2.9 (1.3) | 2.8 (1.1) | 2.9 (1.4) | 2.8 (1.5) | 2.9 (1.3) | 2.9 (1.5) |
|  | *P* |  | .45 | | .96 | | |
| **6.3** | n | 73 | 19 | 54 | 24 | 17 | 13 |
|  | M(SD) | 3.1 (1.1) | 3.2 (1.2) | 3.1 (1.1) | 3.1 (1.1) | 2.9 (1.1) | 3.2 (1.1) |
|  | *P* |  | .31 | | .74 | | |
| **6.4** | n | 73 | 19 | 54 | 24 | 17 | 13 |
|  | M(SD) | 4.1 (1.1) | 4.2 (1.0) | 4.0 (1.1) | 4.1 (0.8) | 4.0 (1.3) | 3.9 (1.3) |
|  | *P* |  | .27 | | .85 | | |
| **6.5** | n | 72 | 19 | 53 | 24 | 17 | 12 |
|  | M(SD) | 2.9 (1.3) | 2.7 (1.1) | 3.0 (1.3) | 2.8 (1.4) | 3.2 (1.4) | 2.8 (1.1) |
|  | *P* |  | .29 | | .59 | | |
| **6.6** | n | 72 | 18 | 54 | 24 | 17 | 13 |
|  | M(SD) | 2.8 (1.2) | 2.6 (1.2) | 2.8 (1.2) | 2.9 (1.9) | 2.7 (1.3) | 2.9 (1.0) |
|  | *P* |  | .27 | | .90 | | |
| **6.7** | n | 73 | 19 | 54 | 24 | 17 | 13 |
|  | M(SD) | 4.0 (1.0) | 4.0 (1.1) | 3.9 (1.0) | 3.9 (0.7) | 4.1 (1.0) | 3.8 (1.3) |
|  | *P* |  | .50 | | .61 | | |
| **6.8** | n | 68 | 15 | 53 | 24 | 16 | 13 |
|  | M(SD) | 3.9 (1.1) | 3.5 (1.2) | 4.0 (1.0) | 3.7 (1.0) | 4.5 (0.9) | 4.0 (1.0) |
|  | *P* |  | .058 | | .053 | | |

Abbreviations: M = Mean, *P = P* value, C1 = Cluster 1, C2 = Cluster, C3 = Cluster 3, LO = Learning Outcome, UE = User Experience, SM = Study Motivation

^a^No single item achieved the total N=74.

^b^*P* values for comparisons between users and non-users are reported as one-tailed tests, whereas *P* values for comparisons within the three user clusters are reported as two-tailed tests.
