# Supplementary material for: Mobile Learning in Medical Education: Quasi-Experimental Realist Evaluation of Usage, Context, and Examination Performance in a Curricular Setting
Source: JMIR Med Educ. 2026 May 21;12:e85892. doi: 10.2196/85892 (PMC13193576; doi:10.2196/85892)
Supplement: Multimedia Appendix 3 [file mededu-v12-e85892-s003.docx]

**Appendix 4A.** Open-ended responses: Perceived strengths of the WueMedica mLearning course, stratified by app usage status and usage clusters.

| **Non-Users** | **Users** | | |
| --- | --- | --- | --- |
|  | **Cluster 1** | **Cluster 2** | **Cluster 3** |
| That it was easy to understand what you were supposed to do. | What I especially liked was the built-in focus on the important facts. I only used Squirrel for a short time, but it really highlighted the key information—things that are frequently asked and relevant in context. | - Links to the Springer chapters - Clear organization of the chapters/microorganisms - Questions well adapted to the teaching and exams | The design, intuitive usability, and leaderboard. |
| Unfortunately, I didn’t use the app. The beginning with the nuts and the texts was a bit too much for me. Other than that, I can't really say much because I didn’t continue using the app. | The feeling of making progress, since you can clearly see how much you’ve already completed. | The comparison. | The leaderboard. |
| Intuitive, fun to use. |  | The increase in points with each review and the competitive spirit. | It’s easy to use and a nice complement to the lecture content. I like that the topics closely relate to the lectures and the relevant general content, and that they’re developed in close collaboration with the instructors. |
| Every topic was included and reduced to the essentials. Not too many questions. | The gamification approach was well done. There were some course contents that weren’t covered in the Anki cards or lecture scripts. What I especially liked was the clear categorization of pathogens—like how the preferred environment was listed for almost every one, which usually takes a lot of effort to look up elsewhere. | Easy to use. | Personally, the app has helped me a lot, as I would otherwise never start reviewing the material at  during the semester. However, the app is so playful and game-ified that I actually used it to repeat the topics  right from the start. I also found the app to be very helpful in understanding the classification of bacteria:  During the final phase of learning, I could always visualize whether the bacteria I was looking for was under, for example, the tab  "gram-positive cocci" or "gram-negative rods". I also found the color coding very helpful, so you knew  while using the app whether you were in the gram-positive or gram-negative section! |
| Unfortunately, I didn’t have time to use it this semester. | The competitive aspect was motivating. | Intuitive, fast, and “convenient.” |  |
| Being able to use it on my phone or iPad and quickly go through something on the go was great. The app gave a good overview of how the bacteria are categorized, with helpful color coding (Gram-negative = red, Gram-positive = blue). Collecting nuts felt a bit like a mobile game, and it was fun to upgrade the learning units from bronze to gold or even higher. | The design is good. The weekly tasks are great. The ranking provides good motivation. | Intuitive interface, mix of images, multiple choice, and text. |  |
|  | The organization of the topics (different bacteria/pathogens) was very clear, and it was great that you could study each pathogen individually instead of in one big, confusing block like sometimes happens with my Anki cards. The questions were really well matched to the lectures, which is why I had such great learning success. | Easy to use, very intuitive, nice extra facts about some bacteria that weren’t in the lecture notes. |  |
|  | Easy to get started, comprehensive. | Designed to be very intuitive; comparing yourself to fellow students motivates you to study regularly. |  |
|  | Short sections. | Playful design with a somewhat competitive aspect; especially finally getting a clear insight into the learning goals and key facts for the exam (compared to other subjects; Microbiology, in my opinion, generally offer excellent teaching). |  |
|  | The points system made it fun to see how I compared to others. | Overview, structure, finding a starting point. |  |
|  | Leaderboard/comparison with others. |  |  |
|  | Follow-up questions on individual topics allowed you to test whether you really knew the key points. |  |  |
|  | Very clear and well-structured. Overall, the learning content was presented in an interesting way. |  |  |
|  | Playful. |  |  |
|  | Learning through play, collecting points, and the ranking system make it even more motivating. |  |  |
|  | System easy to understand. |  |  |
|  | Like a game competition with others—easy to use and accessible anywhere on your phone. |  |  |

**Appendix 3B.** Open-ended responses: Perceived limitations and areas for improvement of the WueMedica mLearning course, stratified by app usage status and usage clusters.

| **Non-Users** | **Users** | | |
| --- | --- | --- | --- |
|  | **Cluster 1** | **Cluster 2** | **Cluster 3** |
| I was unsure whether the course alone would be enough to prepare for the exam. Since you often have little time at the end of the semester, you need to quickly find a resource that can reliably get you through the test on its own. | Show the correct answers right after the first wrong attempt. | Sometimes the sessions are very long, especially at the beginning when your knowledge level is still low, because you only get to see the answers starting from round 3. | It’s a shame there are no explanations for why an answer is wrong. That often leads to just memorizing the correct answer. Also, it would be nice if the virology section were expanded. |
| Being able to see the correct answers immediately… otherwise, I don’t really get much out of it. | Upload higher-quality images, for example, for specimens. | Clicking was sometimes difficult. | There could be better and more appropriate overviews and images for some of the questions—especially those related to microscopy and macroscopy. |
| Dragging the answers into the boxes didn’t have much of a learning effect for me. | Some topics weren’t really useful for exam prep because they seemed designed more for studying for a multiple-choice test. For an oral exam, I would have found Anki decks more helpful since you don’t just recognize the right answers—you have to recall and reproduce them yourself. | Unlocking the learning content only after the lectures was a bit too late for me. Especially with the exam coming up, I would have liked to be able to work on the topics in the app earlier. | Each question includes too much irrelevant information, making it hard to remember everything and causing you to forget the important points. |
| Maybe add typical exam questions for bacteriology, parasitology, mycology, and hygiene as well. | Because of the given answer choices, I got some questions right without really knowing the answers. But in the oral exam, there are no options provided, so the learning didn’t feel 100% suited for that type of test. | The app isn’t suitable for preparing for an oral exam, but it works well for multiple-choice tests. | I found it confusing that the “homework” needed to be completed within a certain time, or it would show you were late, but it never told you how long you had to finish a newly unlocked module. It was frustrating to end up in the “late” category without knowing the deadline. Also, the question type “sort the wrong answers into the trash bin” was hard to answer because answers often fit into multiple categories, making it unclear where to put them. |
| Unfortunately, I didn’t have time to use it this semester. | It’s very time-consuming to have to redo an entire page over and over after just one mistake until you get it right. Overall, I ended up remembering less about the connections and more just which word goes in which blank. After studying with Squirrel, I wouldn’t have been able to explain the bigger picture. I stopped using Squirrel after a few weeks because it was too time-consuming and, for me, not effective enough. | The close texts aren’t really helpful for an oral exam format, and the epidemiology chapters aren’t either in terms of structure. | I think it's a shame that if you get the answer wrong, you don't see the solution straight away, so you might have to do it wrong again. So there is a risk that you will remember the wrong answer. |
| More open-ended questions, since the exam is oral. | I only used it two or three times at the beginning. | The option to mix multiple topics or select larger groups to work on. | You can usually guess the answers in cloze texts. After a short time, you can memorize the answers without understanding them. |
|  | I would have liked to be able to easily look up the things being tested without having to take screenshots of everything... maybe a pre-filled study mode? Or a reference system where you can simply see the info already in the app without having to click through all the quizzes. | Explanations for why answers are wrong, like on Amboss. |  |
|  | I wasn’t sure if all the material was covered and had little time to use WueMedica alongside my current study strategy. | Unfortunately, I didn’t really actively learn the content because my goal was just to finish the unit quickly. I ended up filling in the fields at the end as shown without really thinking it through. Also, I felt the systematics were missing for a long time since the bacteria were mostly grouped by Gram staining and morphology. Similarities in antibiotic therapy, further tests, etc., weren’t included in the initial overview but only listed as facts in later parts. |  |
|  | More questions on virology. | I wish there was a mode similar to Anki, where you could review the bacteria you didn’t answer well. |  |
|  | Better suited for oral exams. This app works more for written ones. Provide fewer suggestions. | Some topics in microbiology were covered less in the course than in the lecture and ultimately in my own exam. But that could also be due to the sheer amount of material and the exam format, where each examiner focuses on different areas. |  |
|  | Collecting nuts feels a bit silly :D | There were some small errors (typos, duplicate words, or wrong answer choices). WueMedica sometimes goes well beyond the lecture content, which was really demotivating at first when I only collected a few nuts... That’s where I struggled most with motivation, especially when questions came up that weren’t mentioned at all in lectures or practicals. Some of the extra info was interesting but definitely not exam-relevant. |  |
|  | Fungi were covered only a little. Virology and parasitology weren’t fully covered. |  |  |
|  | Some parts of the course felt too short, and I missed explanations for why my answers were wrong. Since the topics are quite specialized, it was often very time-consuming to look up the answers myself in books and other sources. |  |  |
|  | Many.  1. It takes too long to work through the individual pages. The questions should be shorter, but there should be more of them, then you can fill the gaps  better.  I would use a maximum of 1 minute per task page as a limit.  2. the fact sheets are great. For the final exam, to get a better overview, it would be useful if you could work through all  profiles together.  3. the repetition requirement is a hindrance to learning. It would be best if you were shown the solutions as soon as you have answered a page and  there is a mistake. I had selected the wrong things from the first repetition, which were then  forever wrong in my memory.  So after the first answer there must be a button to show the solution. For those who like to collect half nuts,  could still be made possible, but I don't think it's the best way to learn. |  |  |
|  | Open-ended questions would be better in some cases since it’s easy to guess otherwise. |  |  |

**Appendix 3C.** Open-ended responses: Additional comments on mobile learning and the WueMedica course, stratified by app usage status and usage clusters.

| **Non-Users** | **Users** | | |
| --- | --- | --- | --- |
|  | **Cluster 1** | **Cluster 2** | **Cluster 3** |
| Unfortunately, I didn't have time to use it this semester | I thought it was very good, even if I didn't use it that much towards the end because I was a bit short of time.... | For the fact that it is still in its infancy, a promising learning idea/help, thumbs up :) | It's really great that you are investing so much time in the project and I'm sure many more subjects could benefit from it! Thank you very much! |
| Thank you very much, I used your offer for the first time this semester and have now passed the exam in my second attempt! | I found it really useful, I was able to learn the bacteria again and check my level of knowledge. Unfortunately,  didn't really help me in the end in the exam, as I was obviously not tested properly by the examiner,  they went on for a long time about little things that I didn't know and so I couldn't show my acquired knowledge (which in my opinion was a lot  , I learned a lot) at all. For my personal level of knowledge and finally for checking what I had learned at  , the app was super useful and will certainly have been helpful for M2 and other subjects in the future :-) | I think it's great that something like this exists. Mibi, in combination with the very good lecture, was much easier for me. | I think the project is great and very helpful. I am sincerely grateful that we received this support! Personally, it helped me a lot and the very large subject of microbiology, mycology, parasitology + virology was brought closer to me in a fun way! The ranking gave me the motivation to stay on the ball and keep going! Thank you very much! |
| / | In the subject area of microbiology, it would have been very helpful to have information only on antibiotics and medicines. | Many thanks for setting it up! A very good and playful addition to the teaching |  |
|  | It's just pointless to study for an oral exam with a question-crossing app... | was great! |  |
|  | Unfortunately, after a few rounds I knew which answers I had to tick or assign without linking this content to a bacterium, for example. As a result, the rounds were often very successful, but unfortunately there was no real learning effect for me. |  |  |
|  | Short question areas.  Classically, as with vocabulary learning, at some point in long-term learning it is important that I can quickly click through the  familiar topics and also take my time with the more difficult points. The important thing is that  progresses quickly enough. In other words, point and click/go and the number of questions per page is not too high. It is better to have more  pages that can be processed more quickly than one full page that takes far too long to process. |  |  |
|  | Thank you for this great learning format! Because I only used it at the beginning, when I hadn't yet dealt with the topics in any other way, the app  didn't help me at all. Terms came up that were not covered in the lecture at the time and  were therefore unclear (e.g. what is the difference between a, b, y hemolysis). Accordingly, it didn't make any sense for me to continue learning with the app  because neither the bacteria nor the properties told me anything at the time  . Later, I had the feeling that I had to catch up on too much in the app, which was too time-consuming for me.  I found that it took a lot of time to have to repeat everything as soon as I got one wrong  . Especially because it was pure guesswork at the beginning...  But I'm generally not a person who likes to learn digitally...  I also know that my girlfriend was very enthusiastic :)  Good luck with your dissertation! :) |  |  |
