# Supplementary material for: Mobile Learning in Medical Education: Quasi-Experimental Realist Evaluation of Usage, Context, and Examination Performance in a Curricular Setting
Source: JMIR Med Educ. 2026 May 21;12:e85892. doi: 10.2196/85892 (PMC13193576; doi:10.2196/85892)
Supplement: Multimedia Appendix 4 [file mededu-v12-e85892-s004.docx]

**Appendix 5.** CMO-configurations.

|  | **Context** | **+** | **Mechanism** | **=** | **Outcome** |
| --- | --- | --- | --- | --- | --- |
| **Non-Users** | - Lower academic baseline (written M1) - Time constraints - Perceived mismatch with oral exam format - Preference for alternative learning tools - Comparable general study motivation | + | - No activation of spaced repetition - No microlearning structure to reduce cognitive load^68^ - No gamification (e.g., leaderboard) to stimulate engagement - No self-assessment feedback - No clearly defined learning goals | = | - Lowest final exam scores |
| **Cluster 1** | - Slightly lower M1 baseline compared to Clusters 2 and 3 - Uniform but low-level engagement throughout the semester - Majority of quests completed only at Level 1 - Perceived time constraints and lack of fit for oral exam - Potentially incomplete coverage of exam-relevant topics | + | - Spaced repetition largely untriggered due to lack of item repetition - Minimal activation of goal-setting, deep learning, and feedback mechanisms - Perceived irrelevance may have reduced intrinsic motivation (strategy-outcome dissonance) - Low efficiency suggests limited self-regulated^[[1]](#footnote-1)^ strategy use | = | - Average performance in final exam - No significant learning gains compared to non-users - App experience rated modestly |
| **Cluster 2** | - Average M1 performance compared to other usage clusters - Intensive app use concentrated in the final phase of the semester - High use of diverse additional learning resources - Likely continuous background learning throughout the term | + | - Activation of strategic control over learning - Frequent exposure to higher item levels → Spaced Repetition - Strong activation of motivational mechanisms (e.g., enjoyment, self-assessment, leaderboard engagement) - Support for goal-setting, metacognitive monitoring, and curriculum alignment | = | - Best oral exam results across all groups - Highest perceived learning outcomes and app experience - Most favorable ratings on enjoyment, retention, and recommendation likelihood |
| **Cluster 3** | - Strongest baseline academic performance (highest written M1 percentile) - Very high early usage, followed by clear drop-off one month before the exam - Lowest number of parallel learning resources reported | + | - Spaced repetition triggered early, but not sustained into exam-relevant phase - Gamification and positive user experience supported early motivation - Strategic disengagement before the exam possibly limited alignment with oral exam demands | = | - Underperformance in final exam compared to baseline |

1. [↑](#footnote-ref-1)
