# Supplementary material for: Mobile Learning in Medical Education: Quasi-Experimental Realist Evaluation of Usage, Context, and Examination Performance in a Curricular Setting
Source: JMIR Med Educ. 2026 May 21;12:e85892. doi: 10.2196/85892 (PMC13193576; doi:10.2196/85892)
Supplement: Checklist 1 [file mededu-v12-e85892-s005.docx]

# CONSORT-EHEALTH Checklist (Expanded, Study-Specific)

| **CONSORT-EHEALTH Item** | **How addressed in this study** |
| --- | --- |
| **Title and Abstract** | The title identifies the study as a mobile learning (mLearning) intervention in medical education. The abstract specifies the quasi-experimental design, voluntary app use, realist evaluation perspective, primary outcome (oral exam performance), and key findings regarding baseline academic performance and usage profiles. |
| **Background and Objectives** | The Introduction provides a rationale for mLearning in medical education, highlights conceptual and methodological fragmentation in the field, and formulates explanatory objectives focusing on learner characteristics, context, and engagement rather than causal effectiveness. |
| **Trial Design** | A prospective, quasi-experimental observational design without randomization or blinding is described. The design choice is justified by the aim to study authentic curricular use. |
| **Participants** | Eligibility criteria, recruitment procedures, voluntary participation, and informed consent are described. The study population consists of undergraduate medical students enrolled in the course. |
| **Setting** | The curricular context, institutional setting, and integration of the mLearning app into routine teaching are specified. |
| **Intervention** | The mLearning app, its pedagogical features (e.g., spaced repetition, feedback), and conditions of access and use are described, without implying causal effectiveness. |
| **Outcomes** | The primary outcome (final oral microbiology exam performance) and secondary outcomes (usage patterns, questionnaire scales) are clearly defined, including timing and measurement. |
| **Sample Size** | All eligible students were invited. No formal sample size calculation was performed, which is appropriate for the exploratory, explanatory design. |
| **Randomization** | Not applicable. The absence of randomization is explicitly stated and discussed as a design characteristic. |
| **Blinding** | Not applicable. Participant blinding was not feasible and this limitation is acknowledged. |
| **Statistical Methods** | Descriptive statistics, t tests, ANCOVA adjusting for baseline academic performance, principal component analysis, and cluster analysis are described. |
| **Ethics and Consent** | Ethical approval by the local independent review board and adherence to GDPR are reported. Written informed consent was obtained from all participants. |
| **Results – Participant Flow** | Numbers of eligible participants, participants included, app users and non-users, and exam participation are reported. |
| **Results – Outcomes** | Exam performance results, adjusted analyses, and heterogeneity by usage profiles are presented transparently. |
| **Harms and Unintended Effects** | No harms or adverse effects were observed. Equity-related concerns are discussed. |
| **Limitations** | Key limitations, including self-selection bias, lack of randomization, and residual confounding, are discussed. |
| **Generalizability** | The Discussion addresses transferability to similar medical education contexts and the need for cross-institutional validation. |
| **Funding and Conflicts of Interest** | Funding sources and potential conflicts of interest are reported. |
