# Supplementary material for: Mobile Learning in Medical Education: Quasi-Experimental Realist Evaluation of Usage, Context, and Examination Performance in a Curricular Setting
Source: JMIR Med Educ. 2026 May 21;12:e85892. doi: 10.2196/85892 (PMC13193576; doi:10.2196/85892)
Supplement: Checklist 2 [file mededu-v12-e85892-s006.docx]

**RAMESES-II reporting checklist for realist evaluation**

| **RAMESES-II Item** | **How addressed in this study** |
| --- | --- |
| **Title** | The title indicates the use of a realist evaluation approach to examine mLearning under authentic curricular conditions. |
| **Abstract** | The abstract summarizes context, analytical approach, key explanatory findings, and implications. |
| **Introduction – Rationale** | The rationale for using realist evaluation is explicitly linked to heterogeneity in mLearning outcomes and limitations of average-effect approaches. |
| **Initial Program Theory** | An initial program theory describing how mLearning might support learning through engagement mechanisms is articulated. |
| **Context** | Learner characteristics, curricular setting, voluntary use conditions, and exam context are described. |
| **Mechanisms** | Mechanisms are conceptualized as motivational and self-regulatory processes emerging from interactions between app affordances and learner characteristics. |
| **Outcomes** | Academic outcomes, engagement patterns, and subjective learning experiences are reported. |
| **Data Sources** | Multiple data sources are used, including app log data, questionnaires, and examination results. |
| **Analysis** | Analytical steps linking context, engagement patterns, and outcomes are described without over-reliance on CMO jargon. |
| **Refinement of Program Theory** | Findings are used to refine the initial program theory, emphasizing conditional and context-dependent effects. |
| **Strengths and Limitations** | Methodological strengths and limitations are discussed in relation to realist evaluation principles. |
| **Ethics** | Ethical approval, consent, and data protection are reported. |
| **Conclusions and Implications** | Implications for curriculum design, equity, and future mLearning research are derived from the refined program theory. |
